# Do medical specialists cope with stressors through fulfillment of basic psychological needs of self-determination theory

**DOI:** 10.5116/ijme.618a.463c

**Published:** 2021-12-03

**Authors:** Stéphanie van der Burgt, Anne de la Croix, Gerda Croiset, Marike Broekman, Saskia Peerdeman, Rashmi Kusurkar

**Affiliations:** 1 Amsterdam UMC, Vrije University Amsterdam, Department of Research in Education, VUmc School of Medical Scienc-es, Institute for Education and Training, Amsterdam, the Netherlands; 2 Cushing Neurosurgery Outcomes Center, Department of Neurosurgery, Brigham and Women's Hospital, Harvard Medical School, Boston, MA, United States; 3 Department of neurosurgery, Amsterdam UMC loc VUMC, Amsterdam, the Netherlands

**Keywords:** Continuing professional development, coping mechanisms, medical specialist, qualitative research, self-determination theory, work motivation

## Abstract

**Objectives:**

To explore factors influencing work motivation negatively and the role of the fulfillment of basic psychological needs, described by the self-determination theory of motivation, as a possible coping mechanism for medical specialists.

**Methods:**

A qualitative study was conducted in an academic medical center in the United States. Twelve medical specialists from different disciplines were recruited through convenience, snowball, and purposive sampling and shadowed for two days each. Semi-structured interviews were conducted afterwards. Data were transcribed, and thematic analysis was used for coding. Themes were finalized through discussion and consensus.

**Results:**

Medical specialists experience three main themes that are identified as stressors; 1) administrative tasks, so-called "administrative jungle", 2) delays and inefficiencies, and 3) poor patient outcomes. To be able to cope with these stressors, medical specialists construct different copingnarratives. Two coping narratives could be linked to autonomy: a narrative of acceptance and a narrative of reinstating autonomy. One coping narrative could be linked to relatedness: a narrative of relationships. No coping narrative could be linked to competence.

**Conclusions:**

The results indicate that coping narratives about autonomy and relatedness are used to cope with moments of pressure, demand, or difficulty, so that patient care can continue to be the first priority. Becoming aware of these coping narratives, using them and reflecting on one's own can help medical specialists in successfully coping with stressors in their work lives. In turn, this can improve specialists wellbeing and performance for patient care as motivation remains.

## Introduction

Physicians work in a highly demanding environment in which they make decisions that can have life-or-death consequences. In highly demanding (work) contexts (i.e., firemen, police force, army, sports, healthcare), a certain level of alertness and perceived tension is necessary to rise to the occasion and handle challenges.^1^ However, levels of awareness, alertness, and tension that are too high can be called stress. The need to keep up with fast-moving changes in the healthcare system in terms of technical innovations and increased societal demands in terms of social accountability poses an additional stressor for b specialists.^2^ This means that every working day demands continuous adaptation from medical specialists.^3^^,^^4^ Although challenging to measure and quantify, previous research suggests that these developments may erode professionalism, influence the quality of care, and increase the risk of preventable medical errors.^5^ However, keeping pace with changes and developing professionally helps medical specialists to perform optimally enhances the quality of healthcare in general and patient safety in particular, as well as minimizes preventable adverse events.^3^^,^^5^^,^^6^

We examine the link between factors that negatively influence motivation, perceived stressors and coping through the fulfillment of basic psychological needs. The aim of this study was to explore factors in the work environment influencing motivation negatively and the role of the fulfillment of basic psychological needs, described by the self-determination theory (SDT) of motivation, as a possible coping mechanism for medical specialists.

Accordingly, the research questions for the present study were:

1)       What do medical specialists perceive as stressors that influence their motivation in their daily work?

2)       How do medical specialists cope with these stressors?

According to SDT, there are three basic psychological needs; autonomy, competence and relatedness.  Autonomy is having the feeling of choice, volition, and congruence. It is supported in environments that encourage behavior congruent with an individual's values. Meanwhile, competence is having the feeling of mastery and that one can meet expected performance standards. It is supported in an environment in which optimal challenges are present. Finally, relatedness is recognizing and feeling connected to role models and peers. It is also about the feeling of belonging among others. Thus, equally important in relatedness is experiencing oneself as contributing to others.^12^ This basic need is supported when others express care and concern.

The fulfillment of these three needs –all equally important-- is necessary for optimal development of autonomous motivation (AM; doing something out of interest or enjoyment or the appreciation of certain behavior as being personally valuable), continuing growth, resilience, and wellbeing. ^7^^-^^11^ AM is considered the optimal type of motivation and leads to better performance, wellbeing, and resilience.

SDT does identify another type of motivation; controlled motivation (CM), which implies that behavior is driven by the promise of reward or the threat of punishment or by internal pressure, such as feelings of guilt or shame.^7^^-^^9^   Moreover, there is a certain hierarchy in this construct in which both types of motivation (AM and CM) exist at three levels of generality.^18^^,^^19^ At the global level, an individual is seen as having developed a global (or general) motivational orientation to interact with the environment in an intrinsic, extrinsic, or a motivated way.1^8^^,^^19^ Contextual motivation concerns the motivational orientations that individuals develop toward each life context (such as education, work, leisure, and interpersonal relationships).^18^^,^^19^ In this study, the contextual motivation of medical specialists concerns their motivation for medical practice. Situational motivation refers to the motivation individuals experience at a particular moment or in a particular situation—the "here-and-now" of motivation—and it is likely influenced by social factors (e.g., rewards, feedback, constraints, deadlines).^18^^,^^19^ In this study, situational motivation refers to the motivation for the different tasks a medical specialist must handle during a day, e.g., handling patients, doing administrative work, and attending meetings.

Previous research (conducted in diverse professional domains) has shown a clear association between need satisfaction and wellbeing at both the general and situational levels of motivation.^7^^,^^13^^-^^15^ Furthermore, the thwarting of basic needs can lead to defensive or self-protective accommodations that have significant negative consequences for health and wellbeing.^16^^,^^17^ Performance of physicians that are unable to keep up with the constantly changing healthcare environment might diminish the quality of care for patients.

### Coping and resilience

Resilience and coping are constructs that reflect the wish to identify characteristics that predict more positive (or at least less negative) outcomes of stress.^20^ Coping is the process of attempting to manage perceived stress and related negative emotions as they arise.^20^^,^^21^ Resilience concerns the prevention of stress incursion, the likelihood of experiencing stress, and negative physiological arousal.^22^ When people are resilient to stressors, they behave in ways that facilitate wellbeing and greater productivity at work.^20^^,^^23^^,^^24^ Processes for enhancing career and professional development insight might be helpful to increase personal awareness, which may improve physicians' connectedness with others and discover sources for greater productivity, energy or creativity. This increases resilience.^25^ Both resilience and coping are a function of person-related and situation-related factors.^20^^,^^26^

Working in high-stakes and complex work environments, such as the healthcare environment, is likely to introduce ubiquitous potential stressors. Stressors deplete adaptation energy. Those with a sufficient reservoir of energy or vitality could be more resilient to demands and challenges, and there is evidence suggesting that active and adaptive coping styles are observed more frequently in autonomous individuals who are more likely to "perceive events as optimal challenging rather than as stressful." An optimal challenging event can be motivating, whereas stress can be perceived as demotivating.^22^ But when is a challenge optimal, and when is it stressful?

An explanation can be found in the 'Goldilocks principle,' which states that there is an optimal level of stress that is "not too much, not too little, but just right." Stress can be positive and necessary, and it can be a state in which the right amount of tension and anxiety leads to higher alertness and excitement. Everyday examples of 'healthy stress' include the nervous feelings one might have before speaking in public or before performing in a sports event. Research indicates that employees operating under an optimal amount of stress learn faster, are more resilient, and are able to juggle multiple tasks.^1^ The optimal level of stress is different for each individual and is dependent on personal resilience and ways of coping with challenges.^1^

## Methods

### Design

A qualitative design was used with a constructivist approach for conducting and analyzing interviews and observations.^30^^,^^31^ In a constructivist approach, there is acceptance of reality and meaning as relative, produced through the interaction between the researcher and the researched, acknowledging the subjectivity of the researchers producing accounts of a social phenomenon.^30^^,^^31^

### Participants and setting

In this study, our definition of a medical specialist is a physician who has completed specialty training. Twelve medical specialists from several disciplines were recruited through convenience, snowball, and purposive sampling. By including different disciplines in this study, we aimed to look at commonalities between different specialties for transferability. (i.e., applying research findings from a particular study to a similar setting).^27^ Snowball sampling was done by asking the participants to suggest their peer specialists for participation. We continued sampling until sufficiency of the data was reached, i.e., sufficiency for gathering the appropriate information to answer the research question.^28^^,^^29^ Data saturation was reached when no new themes or topics were discovered from the data.^28^^,^^29^ This study was conducted in a tertiary academic health sciences center in Boston, MA, USA, a major teaching hospital. Furthermore, it is a research institution in which education, research, publishing, mentoring, and administration are also parts of the specialist's job.

Ethical approval was obtained from and granted by the ethical committee of the hospital. Participants were asked for informed consent and were explained that participation in the study is voluntary, there is a guarantee of confidentiality and anonymity, and non-participation will not cause them any harm. Informed consent was gathered from all participants on paper prior to conducting the observations, acknowledging the anonymized use of their statements in this study. However, informed consent was with minimal disclosure (offering generic rather than specific study information to help minimize the observer effect in field research) to prevent participants from altering specific behaviors.

### Data collection

Data in this study is collected in two ways: (1) through close observation of each specialist for two days in their normal working environment, (2) by interviewing them about their motivation during a working day. Direct observations were conducted by shadowing the participants for two days each to observe the changes in their situational motivation (which can change multiple times daily, as this is the motivation for different tasks within their job). The observation started from the moment the medical specialists entered the hospital and ended when they left the hospital at the end of the day. The goal of the observations was to unravel demotivating factors and ways in which specialists coped with them during a working day. SB observed what happened to the situational motivation of medical specialists and whether an event, activity, or situation was motivating or demotivating. Brief notes were taken during the observations, and extensive field notes were written up immediately after each daily observation to create a thick description.^30^^,^^31^ Observation days were purposively sampled to represent an average day in which the medical specialists had various tasks related to patient care, education, and research. All specialists stated that the observation day was representative of a typical workday.

The interviews were semi-structured and conducted at the end of the second observation day to find links among de-motivators, stress, and coping and as member checking. To aid this interview, we asked them to draw a graph of their motivation during the day and used it as a stimulus to recall the day's events. When asking medical specialists about factors negatively influencing their motivation, rich answers were stimulated by looking at their self-drawn motivation graphs from the observation days and asking about their highs and lows. An overview of the starting questions for the interview can be found in the supplementary file. At the end of the interview, we would ask the specialists how they would change their work environment if given the opportunity to create the ideal environment.

### Analysis

Data collection (observations and interviews) and analysis took place concurrently, with each analysis informing the other. Data from the observations and interviews were transcribed and open coded in a constant comparative manner by two researchers. To explore and categorize the stressors in the specialists' narratives, we used content analysis. The second round of analysis was added, in which we studied ways in which specialists' cope in their narratives. This second layer of content analysis was guided by the three basic psychological needs (autonomy, relatedness, competence) as sensitizing concepts. Whenever there were differences in the open coding, these were discussed until agreed upon by both researchers. Through selective coding and iterative discussion, the research team came to a consensus about the themes and coping narratives.

### Reflexivity

The research team in this study consists of one sociologist, one linguist, and four medical doctors, of whom two are clinical specialists. The interpretation of the research findings is balanced as a result of the research team members' different analytical perspectives. One researcher, SB, was responsible for collecting data from all participants.^30^^,^^31^ This was done to enhance the richness of the data, as each observation considered the context and that the analysis of human behavior may be informed by observing previous contexts. We were attentive to the potential for power differentials and worked to mitigate them wherever possible. Because SB is a sociologist, she is an outsider in the health care work environment. She was thus able to uncover what insiders cannot, as they take everything to be normal or as it is. SB being a sociologist or rather an outsider to the work environment made the specialists open up to her.  The downside of being unfamiliar with the healthcare culture and the American culture is that we might not understand or miss some cultural aspects. However, the clinical specialists in the research team, MB and SP, added the perspective of the work floor into this study. As the other authors have more distance from the daily practice of medicine, they ensured that conclusions were not drawn too prematurely and were grounded in the data. Moreover, MB was also working in the American hospital so she could pick up any cultural aspects that we might have missed. RK is specialized and experienced in studies using SDT, whereas AC has expertise in qualitative research, resulting in a balanced view of the data and the theoretical lens we applied. Through regular group discussions, also in research meetings of the department of research in education, VUmc School of Medical Sciences, Amsterdam, the Netherlands, findings and interpretations were checked for consideration of our own contribution to the research process. Thus, to enhance the trustworthiness of our findings. The first author kept memos of all discussions and steps in the analytical process.

## Results

### Descriptive findings

Twelve medical specialists were observed for two days each and then interviewed. This resulted in approximately 28 hours of observation and around 40 minutes of audio interview per medical specialist. The medical specialists who engaged in this study were neurosurgeons, general surgeons, anesthesiologists, neurologists, and ENT surgeons. Eight specialists were males, four were female, and the average age was 48 (39 - 55) years.

A common view amongst the specialists was that they enjoy the challenges they face every day. They like putting together the puzzle pieces regarding the right diagnosis and treatment for each patient, as illustrated by the quotes below.

"What I like the best is the challenge of figuring out what is wrong with someone, the detective work almost to figure it out, and then the plan and carrying out the plan and seeing that it has worked out." (Medical specialist no. 5)

"I mean, it's the fun part; if you end up at a program like this, then you are the kind of person who enjoys that as a challenge and enjoys being surrounded by really competitive and great people, cause it makes you better, I think." (Medical specialist no. 2)

The challenge is described as 'fun', and the content of their work seems to motivate the specialists. However, there are also aspects that the specialists considered demotivating, which we discuss below.

### What medical specialists talk about: stressors and demotivation

Different issues were described that negatively influenced motivation. We grouped them into three themes linked to the basic psychological needs autonomy, competence and relatedness. These factors are seen as stressors that occur during the day or develop over time in the system. They are displayed in Table 1 with quotes from medical specialists or descriptions of situations that show this demotivation. In a previous study of specialists' motivations, we found that interaction with colleagues (relatedness), being able to organize one's own time (autonomy), and teaching and patient contact enhance autonomous work motivation. However, a lack of knowledge of computer-related issues that impede patient care causes feelings of incompetence and worsens the quality of work motivation.^32^ We have further investigated these findings in the present study and expanded on them.

Although it is common in SDT to call factors that do the opposite of motivating demotivating, the participating medical specialists did not seem to recognize this terminology. When an explanation of the meaning was given, most medical specialists argued that they would call this demoralizing, as shown in the quote below.

"What do you mean, demotivating? [explanation by interviewer]. Oh, demoralizing? Yeah, okay, that is a good question. That is the reverse question." (Medical specialist no.4)

A similar semantic discussion about terminology occurred when using the term 'stress' in the interviews:

"I think the vast majority of the stress that I feel is eustress. It is a positive pressure inherent to the work that I do. The other thing is distress." (Medical specialist no. 1)

**Table 1:** An overview of demotivating factors

Demotivating factors		
Delays and inefficiencies	Administrative jungle	Poor patient outcomes
· Any issue or matter that causes a delay in medical specialists' schedules was considered demotivating. Specialists referred to these issues as "*stuff that slows you down.*" Examples that were mentioned were: meetings, turnover time, or general logistics in patient care (i.e., patients not showing up or when the specialists were not called at the point when a patient shows up to the clinic). These events decreased their feelings of autonomy because they have no control over the events or their schedule, nor do they feel like there is enough time to manage all the different tasks, to the point where even negative effects on their emotional and physical wellbeing are mentioned: · *"There are days where it is just one struggle after another, where I feel I do not have control of the practice that I would like to have." (Medical specialist no. 9)* · *"When you do surgery, there is that time in between surgeries. It is a very long time, and things do not really move along. You spend a lot of time waiting for things to happen. You can do other things, like your paperwork, but it does make for a long day. That part of waiting around, doing paperwork, these inefficiencies, that part is no fun." (Medical specialist no. 10)* · *"Sitting in meetings, all meetings. All meetings that are not directly related to my research or patient care I find very irritating. It gives me headaches." (Medical specialist no. 1)* · *Demanding patients can also be categorized under this theme. The specialist seems to experience a loss of autonomy, as the patient did not take their advice (in this case, a referral to a different specialist). There also seems to be a lack of relatedness, as the patient and specialist find it impossible to come to a shared decision. Finally, there seems to be a lack of competence, as the specialist does not know what to do next and cannot provide the care they would like to provide.*	· Administrative tasks are considered a burden. The bureaucracy and "daily grind" of administration, phone calls, a focus on patient satisfaction, and measurement take away time from patient care—what specialists actually want to do. Specialists refer to "the paper jungle," which decreases their feelings of autonomy. · "I do not feel like I am doing a lot of the stuff that I was hoping to do when I started medicine." (Medical specialist no.7) · "The demoralizing aspects are the amount of administrative and bureaucratic…the burden that does not add anything to the quality of our outcomes. You are spending all this time looking at computers and satisfying what the computers want you to do." (Medical specialist no.12) · The administrative activities are not seen as relevant to the ability to provide the best quality of care. Some of these tasks may be irritating or start out irritating, but when dealing with those days, they become demotivating or even demoralizing.	· As mentioned before, medical specialists enjoy the challenge and the puzzle of treating patients. However, when their decisions regarding patient treatment lead to poor outcomes, it is considered highly demotivating. There is a feeling of a loss of competence, as apparently, the specialist was unable to complete this challenge and rise to the occasion. All specialists stated this same feeling: · "The hardest part is, sometimes, we guess wrong, and we end up not giving people a successful procedure, or it just does not do what we want to do, or we need to revise it, and they have to go through more stuff." (Medical specialist no.8)

Table 1 shows that demotivating factors are main tasks and organizational processes that distract from patient care or that compromise the quality of care. Using medical specialists' experiences of demotivating factors made it easier to talk about the ways they try to cope with the experienced stressors and de-motivators.

### How medical specialists talk about it: coping narratives

Despite demotivating factors, specialists find ways of coping with stressors. In the interviews, we found different ways in which specialists talk about the demotivating factors and their professional life. The way these stories or narratives were framed seemed to help specialists cope with their work challenges. The participants seem to handle stressors by constructing and telling themselves 'coping narratives' that justify the situations and behaviors, as the quote below indicates:

"Usually, I get pretty angry immediately, but I let it kind of sit, and I think about it, and I can usually talk myself out of being upset." (Medical specialist no.6)

**Table 2:** Overview of coping narratives

Coping narratives	
Narratives about the fulfilment of autonomy	Narrative about the fulfillment of relatedness
1) The narrative of acceptance We recognized narratives of acceptance, in which specialists coped with daily stressors by accepting them and accepting that they have no influence over the stressors. 2) The narrative of reinstating autonomy A second narrative that we recognized was one of control. Medical specialists actively try to gain control over their tasks and daily schedule despite the 'administrative jungle' and other threats to their professional autonomy.	3) The narrative of relationships The third narrative is about togetherness and relationships, both at work and at home.

In this section, we describe these coping narratives, which are also displayed in Table 2, and support them with quotes or comments. The coping narratives used by the medical specialists are linked to autonomy and relatedness. All narratives can be used for coping with all types of demotivating factors.

### Fulfillment of the three basic psychological needs

#### Autonomy

### The coping narrative of acceptance

We recognized narratives of acceptance in which specialists coped with daily stressors by accepting them and accepting that they themselves have no influence over them. Some specialists emphasized that there is no choice but to accept the way the system works. Acceptance is necessary because the patients need to be taken care of, which is important to them, as the quote below shows.

"It is just how it works, it is not really a choice. The clinical things just have to be taken care of. The patients are here, they have to be taken care of. They always have the priority over everything else." (Medical specialist no. 9)

Therefore, the flaws or stressors in their daily work lives are justified and accepted by emphasizing that patient care that must go on. One specialist commented that he just ignores 'the background issues,' which in this case is a synonym for stressors, as these are less important to him than doing what he loves: patient care. The quotes below are examples of what medical specialists say about this:

"Then, I just kind of see my patients and take care of my patients and try to ignore all the background issues." (Medical specialist no. 11)

"I love doing surgery, and I love taking care of the patients, so I just say, "Okay, well I will just ignore the background and kind of move forward." (Medical specialist no.2)

Another way to achieve acceptance is to wait for a solution for flaws in the system or to trust that a solution is being devised. In other words, accepting the current situation is justified by emphasizing that a solution to the problems is in the works somewhere:

"I used to get worried about everything, and now I kind of just trying to help out in whatever way people ask me to. Adding on patients, doing things that help our division, and realizing that the process is in the works, it's going to happen and just kind of riding the wave until it gets solved." (Medical specialist no.12)

The quote shows that specialists describe 'ignoring' and waiting for solutions as mechanisms to cope with stressors. Within the coping narrative of acceptance, the specialists do not see themselves as having an active role in changing the situation or the system. Complaining is explicitly mentioned as being ineffective, as it "really does not do anything. So you just need to figure out a way around it then." The demotivating factors are seen as an established fact; they are accepted as part of their professional reality. By providing themselves with these different ways of accepting and telling themselves that it is in the best interests of their own professional priorities (i.e., patient care), the specialists cope with the stressors. Accepting the situation is framed to be essential to the greater good in this narrative. At the same time, they are seeking to fulfill their need for autonomy. When they feel in charge of a situation or that they have a choice in it (the choice, in this case, being acceptance), it satisfies their feeling of autonomy. Thus, coping involves moving from the perception of a loss of autonomy in the situation to gaining autonomy by telling oneself that it is for a greater cause and one's own choice. This is clearly visible when stressors are reframed as 'silly things' when specialist no. 1 was asked what s/he would change in their work environment if they would have the opportunity to create their ideal environment:

"Okay. See where it is at. I have no idea. I am not sure… I mean, I am very fortunate. Maybe a whole bunch of silly things, which are silly like maybe having your offices closer to the operating rooms but no." (Medical specialist no. 1)

Overall, the specialists reported little they would want to change. On the contrary, they are actually very pleased with and grateful for what they already have. For example:

"No. I am very.. of course, this is a system that has worked out that there is a culture of excellence around here that permeates this place. It is a pretty nice place to work. I would like to get paid twice as much, of course who would not." (Medical specialists no. 7)

In other words, the specialists accept that no opportunities or possibilities are seen to change anything and thus talk about being grateful and happy or pleased with the situation as it is.

### The coping narrative of reinstating autonomy

The second coping narrative that we recognized was one of reinstating autonomy, in which medical specialists actively try to find autonomy over their tasks and daily schedule. Specialists argued that they undertake specific steps to ensure they can schedule the day exactly the way they want it to be. This may involve changing appointments or not volunteering for any extra work, as the quotes below state.

"When it has to do with patients and optimizing their outcomes, optimizing their procedure and their outcome, that is my priority, so I do not care if I have to cancel patients. I do not care if I have to move patients; that is the priority for that day. I need my scheduling the way I want it because I do not want to feel rushed. I do not want to feel like the patient, their care, is being compromised because I need to see a certain number of patients." (Medical specialist, no. 4)

"I do not volunteer much to add extra patients or (administrative) tasks. It is a strategy for protecting myself from extra work that is not really value adding to have a physician doing that work." (Medical specialist no. 3)

The quotes not only show medical specialists taking action to reinstate autonomy, but they also show that the action is mainly taken when it comes to patient care and wanting to provide the best care possible, which is clearly their priority. In other words, it seems like taking action and reinstating autonomy to be able to handle stressors is most important for fulfilling priorities and for being able to provide the best possible care. By taking these actions to reinstate autonomy, specialists search to fulfill their need for autonomy, but it may also be for competence. Because of their actions, they feel competent in caring in the best way possible. When it comes to extra work, the specialists stated they do not volunteer much for these tasks or they "try to build in some space to their schedule in the clinic so that it is not full" as a protection mechanism for too much work. Hence, it seems they are trying to find a balance in doing what needs to be done and having the time and opportunity to do these things right—they are inherently trying to reinstate autonomy again. Because the specialists actually try to take reinstating autonomy l again by undertaking an action, they gain but also must feel more autonomy and competence. By finding their own personal ways of action taking, they feel more autonomous.

Parallel to these coping narratives about the fulfillment of autonomy and relatedness; medical specialists state the importance of another factor: age. As specialists grow older and more experienced in their work field, they feel inherently there is more fulfillment of autonomy. When they were younger, they had to put in the hours, the effort, and the work to prove themselves and make a name for themselves.

"I did make a choice to go slower or easier with my days and work, but I could. I could because people wanted me enough. When you are young, people do not want you; they just want somebody to do the work. And the only way that you get it is by being there or by being willing or by being the person who is happy to show up at two o'clock in the morning to fix the problem, right? That is the only way. Eventually, people say, "No, I do not want anybody; I want you." That is different, but a huge amount of investment to get it that way." (Medical specialist no. 2)

"My old life was... I was full-on. I was doing a big clinical volume, teaching, research, and trying to live a family life with three kids. That and then any little thing that adds or screws up your schedule, even then, I would over schedule and I would sort of say, come on, come on, come on. Let's go, hurry up, because if we do not get started now, I am never getting out. That is not how I practice anymore. I am done. I am not going to stay anymore. It is somebody else's job." (Medical specialist no. 4)

The older specialists clearly state that they practice differently now in their positions because they feel it is possible to do so. They recognize these situations in their younger colleagues and that it might not be the healthiest situation to work in, but they also think it is the only way to get ahead in this work field.

"Because he is really good at what he does, but he is in the big pond here. He is trying to prove that he is as good as he thinks he is, and he is trying to establish his practice, establish his academic position. He is supervising and mentoring a whole bunch of students and things. He works in two hospitals, really crazy. And he has even got a family with young children." (Medical specialist no.2)

In other words, the lack of autonomy that younger specialists experience is caused by them feeling that there is no other way than to do the things that need to be done. Things get better as they age, because they will have proven themselves and gained some authority, which gives them the power to decide to do more of the things they love (for example, more surgery) and schedule their time as they like it.

### Relatedness

#### The narrative of relationships

The third narrative is about relationships. The specialists state that they feel there is mentor support in which the older, more experienced specialists guide the younger, less experienced ones. This is seen as useful:

"We have great people around us. We have a lot of great older surgeons that are very mentor kind of people who talk to you. Encourage you. That kind of support is helpful." (Medical specialist no. 1)

However, they all state that togetherness is generally lacking in their work environment:

"We are very busy, and we just come together, and it is not like we are sitting around. We do not sit around and chit chat." (Medical specialist no.7)

They do mention having a desire for more cohesiveness and support from peers, as they feel work would be much more enjoyable:

"I do wish that our department was a little more cohesive, our division." (Medical specialist no. 6)

"I think you would be much more of an isolationist and even much more protective of yourself and your interests. And you enjoy your work a lot less. You are dealing with also all the negative lack of peer support that goes along with it." (Medical specialist no.5)

Some of the specialists reported that it does not feel safe to talk to peers about stressors or struggles. It is a competitive environment, and it does not allow for doubt or an inability to handle the challenge:

"I think it is very important to be able to vent about the day to your colleagues, but we won't do that because then we have to admit that we were not up to the challenge to another colleague." (Medical specialist no.6)

"I think the expectation is that we are going to talk about it. How you perceive safety is a little beside the point because it is going to happen. It is like giving a speech in class. You feel like it is not safe, but it is the assignment." (Medical specialist, no. 1)

As the last quote illustrates, there are moments in which the specialists talk to each other about difficulties or complex cases/challenges, but only because it is expected, obligatory, and part of the job. It seems this commitment can be a stressor itself. Although the specialists clarify that there is a need for more support and relatedness, at the same time, they suggest it is hard to find a way to arrange this:

"Honestly, my answers are really all over the place because I do not fully understand it. I do not fully understand what I need to do to keep happy in this job, whether it is just being with people and doing the stuff that I enjoy doing, but for me, my personality is more interesting. That kind of stuff, for me, is definitely a lot more personal and solo. It would be hard for me to be in a large group of doctors talking about this stuff without it becoming a gripe fest about specific things, like the computer system or whatever people are complaining about the most. I try to take care of myself the best I can, but I do not really know what the answer is for doing more as a group or as a profession." (Medical specialist, no.7)

The medical specialists seem to believe it is impossible to arrange some sort of support system with which they would feel comfortable and that they do not feel they have the right tools or abilities to take action. Because these specialists are unable to find support or togetherness in their work environment, they all commented that personal relationships are important. The personal relationships are the ones they use to negotiate stress:

"Family and friends are who I talk to. I do not talk to my peers here." (Medical specialist, no.9)

"I have a great marriage; it does not solve everything, but it certainly makes it easier to negotiate all stressors." (Medical specialist, no. 1)

"This foundation of primary relationships puts you on a steady foundation so that when you can counter stressors at work, you are much more likely to negotiate them in a positive way versus getting upset, yelling, becoming irritated, and exhibiting signs of distress." (Medical specialist, no.10)

These quotes show that the support and relationships that the specialists get at home or in their private lives provide them with some resilience to cope with the daily stressors they experience in their work.

Age is also a factor when it comes to relatedness. Medical specialists argued that personal relationships, in particular, have to suffer from this culture or system, in which they have to work hard, be competitive, and put in the hours.

"Well, you disappoint a lot of people. That is one of the problems, and that is why relationships fail. Professional ones do not, but personal ones do." (Medical specialist no.6)

"I was working 120 hours a week. Never saw your family. Period. Go to work at five in the morning, come back at eleven or twelve, every day, including Sunday. For years. But looking at it now, it is great, right? I get to do all these cool things in this cool place. But there is a lot of sacrifices that go on in there, a lot. Looking through your family pictures and not being in them." (Medical specialist no.2)

Again, as mentioned earlier, it seems like medical specialists do not feel like they have the opportunity or tools to change this.

"And that can take its toll unless you put limits on yourself and your practice. But those are very hard for people like us to do because we are people who do not know limits. We do not know physical limits. We do not; well, we try not to know intellectual limits." (Medical specialist no.8)

Because with age comes more autonomy, the priorities of medical specialists change, and they develop a need for a different kind of work-life balance. Personal priorities can have a place in this work-life balance, which seems an important realization to experience fewer stressors in general.

#### Competence

The coping narratives that we identified involve autonomy (the narrative of acceptance and the narrative of control) and relatedness (the narrative of togetherness), not competence. The specialists do not talk about being or feeling competent. They describe themselves as high-performing individuals who learn on the job. Therefore, it seems the feeling of being competent is already there, and there is no struggle or searches to fulfill the need.

## Discussion

The present study was designed to unravel stress factors for medical specialists and understand how the specialists cope with these stressors in their daily work. Three main themes were identified for stress factors. The themes were; delays and inefficiencies, the administrative jungle, and poor patient outcomes. These are in line with stressors identified previously among medical specialists.^6^^,^^25^^,^^32^^-^^35^

The themes of delays and inefficiencies and the administrative jungle both seem to frustrate medical specialists' feelings of autonomy. Specialists indicate that they do not perceive autonomy in making their own schedules. In addition, delays and inefficiencies seem to be a distraction from specialists main tasks, which is caring for patients. As mentioned before, patient care is what motivates medical specialists the most, as it combines the fulfillment of all three basic needs: fulfillment of autonomy, as the specialist can decide on the treatment; fulfillment of competence, as they can provide the best care and fulfillment of relatedness, as they can relate to their patients.^33^ From our results in this study, the opposite, poor patient outcomes, is shown to be a stress factor that can take away from fulfilling medical specialists' need for competence, as they feel or were unable to provide the necessary care to a patient. Furthermore, when discussing stressors or factors influencing motivation negatively, we identified some semantic issues, as the medical specialists did not seem to recognize the term demotivating. When our definition was explained to the medical specialists, they recognized the concept as demoralizing.

Regarding the search for stressors, this study aimed to determine how medical specialists cope with stressors during their daily work. The findings highlight narratives that specialists construct when asked about methods of coping. Three coping narratives were found, two about a search for the fulfilment of autonomy and one about the fulfilment of relatedness. In the first narrative, which we call the narrative of acceptance, medical specialists frame stressors and de-motivators as established parts of their daily reality and accept them as they are. Acceptance might be a successful narrative to sustain motivation. However, it is also possible that acceptance merely provides a short-term coping mechanism, and another solution is needed in the longer term. The second coping narrative might be a more long-term one. The narrative of reinstating autonomy demonstrated that medical specialists take action to reinstate autonomy of their time and responsibilities, especially when it comes to protecting their patient care duties. The third one, which we called the narrative of relationships, showed a lack of relatedness at work. This fulfillment of relatedness is sought and found at home and within their private lives and relationships.

The medical specialists also explained that there are two types of stress, "eustress and distress". This separation between positive and negative differences in stress fits within the Goldilocks principle, as discussed previously.^1^ This principle states that there should be a balance between good (positive) stress, which provides alertness and focus, and bad (negative) stress, which provides negative outcomes. This balance is different for each person, but the rule of thumb for the number of stressors is not too much, not too little, but just the right amount. The coping narratives that specialists construct are the mechanism to cope with the distress and to find and keep the balance between eustress and distress. Therefore the coping narratives could help specialists in performing as optimal as possible. When medical specialists perform as optimal as possible, a higher quality of health care can be obtained.

Another important factor that plays a role in coping with stressors, which was age. Their autonomy grows with age. Perhaps older medical specialists are more prone to telling the narrative of control because they experience more autonomy. They might see or feel more opportunities to take actual control, and, in turn, feel even more autonomous.

Specialists construct these coping narratives to justify their situation or struggle, which is how they consciously or unconsciously cope. As a final question, we asked the specialists how they would change their working environment if they had the opportunity to create an ideal workplace. Surprisingly, they were not able to come up with any suggestions. Most of the specialists were humble, grateful, and happy with how the hospital work is organized. This might also be caused by the hidden culture of working in a very prestigious University-affiliated hospital, in which one can only feel grateful for having the opportunity to work in such a place and not critique it. Still, constructing one of these coping narratives enables specialists to cope with the stressors and de-motivators they experience daily.

### Implications for practice and research

The results of this study can guide medical specialists toward successfully coping with stressors in their work lives. It might be helpful for specialists to have a mentor or coach in helping them reflect on a scenario and then have the mentor lead them through it. In this way, they could become more aware of their coping narratives. A mentor could help them find ways to take action to improve stressful or demotivating situations, as an individual is only satisfied as his/her energy is put towards the things that lie in their circle of influence as opposed to their circle of concern.^36^ Circle of concern includes all concerns a person has which may be in his influence and also those that are not under his influence, but maybe under the influence of the department, institution, profession or Government. Within this circle is the smaller circle of influence, which includes the concerns that can be influenced by this person. Specialists should primarily focus on changing concerns that are within their circle of influence, which is seen in the narrative of reinstating autonomy. The narrative of acceptance is seen in the issues that are in their circle of concern and not in their circle of influence. But this does not mean that the specialists cannot do anything about these issues. Changes in such concerns need to be initiated or channeled through people in the organizations responsible for them, i.e., people who do have these issues in their circle of influence. Specialists can work towards expanding their circle of influence by organizing mentors for themselves who do have the power to change things or can coach them on how to get things changed. Doing so helps in replacing the narrative of acceptance with the narrative of reinstating autonomy through making use of the narrative of relationships at work. This connects specialists with colleagues who have a bigger circle of influence than themselves. As recently, the focus has shifted from how organizations can motivate employees from the outside to instead on how they can effectively foster and support high-quality autonomous motivation within employees. There are also implications for the department/organizational level.^37^ A support system could be set up at the department level, making it easier for specialists to find the right person to support them or mentor them. A second implication is to make medical specialists as autonomous as possible and to strengthen their relatedness at work. Additionally, more non-physician medical providers should be placed within the departments to assist with or take over administrative tasks or even to take these tasks away from the specialists.

Further research is necessary to find the right ways of helping specialists determine their own coping narratives. This study led us toward narratives that seem promising in finding out more about the professional lives of physicians. We would recommend further work in this area in accordance with those advocating for narrative research in medicine.38 It would also be helpful to undertake similar research projects in other high demanding fields (i.e., firemen, police force, sports, army) to determine whether the same coping narratives are found.

### Strengths and limitations

To our knowledge, it is unique combining the basic psychological needs, stressors, and coping in one research. Also, relatively little qualitative work has been done using SDT, especially in health care and education. The coping narratives we found, based on autonomy and relatedness, might be the start of a new and qualitative approach to research on work motivation.

A potential limitation is the sampling method. Using snowball sampling could have led to a more motivated sample of medical specialists than the general population. This could paint a more positive picture of the motivation of medical specialists. However, if we indeed have more motivated specialists in our sample and still found these demotivating factors for medical specialists to do their work at an optimal level, we could say these factors are highly important. This may be even more important for less-motivated specialists, and these factors may demotivate them even more. Furthermore, if the specialists in this study were the most motivated, we likely also found the most helpful coping narratives. The study is conducted in the context of an academic environment and involves the participation of only a selected number and types of specialties. Therefore, the findings may not be applicable to other clinical practices and/or other specialties. Finally, a strength of this work has been the combination of several types of data to get a rich and deep understanding of the phenomenon under investigation.

## Conclusions

Medical specialists experience many different factors that influence their motivation negatively. Patient care was reported to be the most motivating factor. However, other tasks and responsibilities identified as delays and inefficiencies, the administrative jungle, and poor patient outcomes continuously take away time from patient care. To cope with the factors that influence their motivation negatively, medical specialists construct narratives. Autonomy and relatedness play a role in the way medical specialists talk about coping with moments of pressure, demand, or difficulty. Specialists either try to fulfill their autonomy by constructing a coping narrative of acceptance or the narrative about reinstating autonomy, so that patient care can continue to be their first priority. The third narrative of relationships is about relatedness or rather lack thereof. Specialists would like to be more related to their colleagues but do not experience having the tools to organize more relatedness at work. So, they end up looking for relatedness in their personal relationships.

The key to these coping narratives and sustaining motivation seems to lie in the specialists' passion for patient care. The coping narratives may be used consciously or unconsciously; they ensure that issues/stressors are surmountable in the moment and longer-term motivation for medical practice remains. This can sustain medical specialists' optimal performance, which in turn benefits the healthcare quality.

### Acknowledgements

The authors would like to thank all medical specialists who participated in this study.

### Conflict of Interest

The authors declare that they have no conflict of interest.
